# Behavioral Effects of a Chemorepellent Receptor Knockout Mutation in *Tetrahymena thermophila*

**DOI:** 10.1128/mSphere.00182-17

**Published:** 2017-07-05

**Authors:** Dianxiong Zou, Todd M. Hennessey

**Affiliations:** Department of Biological Sciences, University at Buffalo, Amherst, New York, USA; University of Georgia

**Keywords:** GPCR, knockout mutation, behavioral bioassay, chemorepellent receptor, chemosensory transduction, excitability, *Tetrahymena*

## Abstract

A conditioned supernatant from *Tetrahymena thermophila* contains a powerful chemorepellent for wild-type cells, and a gene called *G37* is required for this response. This is the first genomic identification of a chemorepellent receptor in any eukaryotic unicellular organism. This conditioned supernatant factor (CSF) is small (<1 kDa), and its repellent effect is resistant to boiling, protease treatment, and nuclease digestion. External BAPTA eliminated the CSF response, suggesting that Ca^2+^ entry is required for the classical avoiding reactions (AR) used for chemorepulsion. A macronuclear *G37* gene knockout (G37-KO) mutant is both nonresponsive to the CSF and overresponsive to other repellents such as quinine, lysozyme, GTP, and high potassium concentrations. All of these mutant phenotypes were reversed by overexpression of the wild-type *G37* gene in a *G37* overexpression mutant. Overexpression of *G37* in the wild type caused increased responsiveness to the CSF and underresponsiveness to high K^+^ concentrations. Behavioral adaptation (by prolonged exposure to the CSF) caused decreases in responsiveness to all of the stimuli used in the wild type and the overexpression mutant but not in the G37-KO mutant. We propose that the constant presence of the CSF causes a decreased basal excitability of the wild type due to chemosensory adaptation through *G37* and that all of the G37-KO phenotypes are due to an inability to detect the CSF. Therefore, the G37 protein may be the CSF receptor. The physiological role of these G37-mediated responses may be to both moderate basal excitability and detect the CSF as an indicator of high cell density growth.

**IMPORTANCE** Although many single-cell eukaryotes have served as classical model systems for chemosensory studies for decades, the major emphasis has been on chemoattraction and no chemorepellent receptor gene has been identified in any unicellular eukaryote. This is the first description of a gene that codes for a chemorepellent receptor in any protozoan. Integration of both depolarizing chemorepellent pathways and hyperpolarizing chemoattractant pathways is as important to chemoresponses of motile unicells as excitatory and inhibitory neurotransmitter pathways are to neurons. Therefore, both chemoattractant and chemorepellent pathways should be represented in a useful unicellular model system. *Tetrahymena* cells provide such a model system because simple behavioral bioassays, gene knockouts, biochemical analysis, and other approaches can be used with these eukaryotic model cells. This work can contribute to the basic understanding of unicellular sensory responses and provide insights into the evolution of chemoreceptors and possible chemorepellent approaches for preventing infections by some pathogenic protozoa.

## Abstract (precis)

Although many single-cell eukaryotes have served as classical model systems for chemosensory studies for decades, the major emphasis has been on chemoattraction and no chemorepellent receptor gene has been identified in any unicellular eukaryote. This is the first description of a gene that codes for a chemorepellent receptor in any protozoan. Integration of both depolarizing chemorepellent pathways and hyperpolarizing chemoattractant pathways is as important to chemoresponses of motile unicells as excitatory and inhibitory neurotransmitter pathways are to neurons. Therefore, both chemoattractant and chemorepellent pathways should be represented in a useful unicellular model system. *Tetrahymena* cells provide such a model system because simple behavioral bioassays, gene knockouts, biochemical analysis, and other approaches can be used with these eukaryotic model cells. This work can contribute to the basic understanding of unicellular sensory responses and provide insights into the evolution of chemoreceptors and possible chemorepellent approaches for preventing infections by some pathogenic protozoa.

## INTRODUCTION

Unicellular eukaryotic organisms such as *Tetrahymena* integrate sensory information in a manner that is similar to that of neurons in higher organisms. In ciliates such as *Tetrahymena* and *Paramecium*, chemorepellents generally cause depolarizations (1, 2) and chemoattractants provide hyperpolarizations (3). In general, membrane hyperpolarization causes faster forward swimming while depolarization slows forward swimming (4). If the cell is depolarized past the threshold, a Ca^2+^-dependent action potential will fire and the cell will swim backward because of the increase in intraciliary Ca^2+^ (5). Therefore, swimming behavior can represent the integration of excitatory and inhibitory influences that can culminate in an observable Ca^2+^-dependent event. In this respect, chemorepellent responses are as important to ciliates as excitatory neurotransmitters are to neurons. However, no chemorepellent receptor has been reported in the genome of any unicellular eukaryote.

Many sensory responses in eukaryotic cells are initiated by ligand binding to a G-protein-coupled receptor (GPCR), and several studies suggest that GPCR signaling does exist in ciliates (6, 7). We have identified several putative GPCRs in the Tetrahymena Genome Database (8) and made macronuclear knockouts to try to match a chemosensory GPCR with a physiological function. Our first gene knockout of a putative GPCR (GPCR6; accession no. TTHERM_00925420) resulted in not only loss of chemoattraction but also reduced G-protein activity (9). This was the first example of the effects of GPCR activity on chemosensory responses in any ciliate (9). We also identified another possible GPCR in the Tetrahymena Genome Database (TTHERM_00633170) that we have named G37. In this report, we describe the altered responses of a *G37* gene knockout mutant that we call G37-KO.

We have chosen the ciliate *Tetrahymena thermophila* as a simple model organism for the continuing study of chemosensory responses because of a number of important characteristics. *Tetrahymena* displays recognizable and reproducible changes in swimming behavior when exposed to chemoattractants (9) and chemorepellents (10). The electrophysiological basis of the behavior of *Tetrahymena* is similar to that of *Paramecium*, so swimming behavior changes can be used for estimation of electrophysiological changes (1) and changes in intraciliary Ca^2+^ levels (11). Targeted genetic manipulations, such as gene knockouts, are well described in *Tetrahymena* (12, 13). The cells grow to high densities in axenic cultures, providing sufficient starting material for biochemical analysis.

The basis for a strong chemorepellent response in ciliates like *Tetrahymena* and *Paramecium* is the avoiding reaction (AR) (14). Voltage-dependent Ca^2+^ channels are localized to the ciliary membranes, allowing for depolarization-dependent Ca^2+^ influx into the intraciliary space. Elevation of intraciliary Ca^2+^ above 10^−6^ M causes ciliary beat reversal and backward swimming (5, 15). In a classical AR, the action potential is transient. This causes the cell to swim backward briefly but then resume forward swimming as intraciliary Ca^2+^ levels return to normal. As the cell resumes forward motion, it swims off in a new, random direction. This causes a random change in swimming direction that serves the same behavioral purpose as the tumble response seen in bacterial chemotaxis (16). As in bacteria, direction changes (DCs) are the main basis for the chemorepellent responses of *Tetrahymena*. The frequency of DCs increases as cells approach a chemorepellent gradient, producing many new and random swimming paths. New swimming paths that take cells away from the repellent will contain less AR and straighter forward swimming away from the repellent. *Tetrahymena* will show repetitive AR when it is transferred into a chemorepellent solution from which it cannot escape, and these AR can continue until the cells adapt to it and regain normal forward swimming.

Swimming behavior can also be used to estimate the strength of a stimulus (2). Mild depolarization can lower the forward swimming speed, while stronger threshold depolarization can elicit an AR. Repetitive AR can represent an even stronger stimulus. A very strong, prolonged stimulus can cause continuous ciliary reversal (CCR), and the duration of the CCR is an indicator of how long it takes for the cell to regain basal intraciliary Ca^2+^ levels (17, 18). It has been shown in *Paramecium* that such CCRs can be terminated by a combination of Ca^2+^ channel inactivation (19, 20) and Ca^2+^ removal (18, 21) to enable resumption of forward swimming. Because of the involvement of Ca^2+^ channels in this response, CCR duration has also been used as a bioassay for Ca^2+^ channel function in *Paramecium* (20). It is assumed that similar mechanisms exist in *Tetrahymena* (22).

Prolonged exposure to a chemorepellent can cause chemosensory adaptation, seen as a decrease in responsiveness to that chemorepellent over time. This can be due to either a specific change such as receptor desensitization (23) or more general changes in membrane excitability (17, 24, 25). Cross adaptation to other ligands that use the same receptor or the same sensory transduction pathway can be seen, so this can be used to see if different ligands use the same receptor or sensory pathway (24).

There are many examples of conditioned medium factors (CMFs) produced by eukaryotic unicells during growth, but no chemorepellent receptor has ever been matched to a factor in the growth medium. Such factors could be forms of intercellular communication between these unicells. A CMF and several other secreted factors have been described in *Dictyostelium* (26). Some of these factors are chemorepellents (27, 28), but their receptors have not yet been identified. Intercellular signaling by secreted factors of unicellular eukaryotic cells has been reviewed (29), but no repellents were mentioned. A soluble transforming principle released by *T. thermophila* was found to cause macrostomal transformation in *Tetrahymena vorax* (30), and it was later identified as a compound called stomatin (31). This factor has been described as a chemoattractant (32), but the structure of this compound has not been confirmed and the receptor is not known. However, when we isolated a similar conditioned supernatant factor (CSF)-containing fraction from *Tetrahymena*, we found that it not only had chemorepellent activity but also was not recognized by our new G37-KO mutant.

The objective of the current work was to identify the major phenotypic differences between this G37-KO mutant and the wild type in order to gain insights into the roles of chemorepellent receptors in both the basal and chemosensory responses of *Tetrahymena*.

## RESULTS

### Prediction of the structure of the *Tetrahymena* G37 protein.

**FIG 1:**
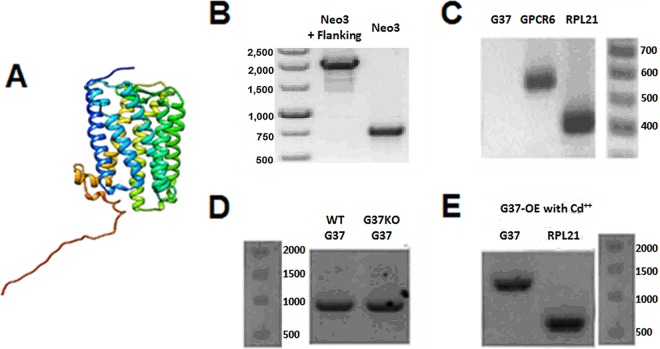
(A) The predicted structure of the G37 protein from the *Tetrahymena* amino acid sequence (TTHERM_00633170) was drawn with the GoModo fitting program (43). The N terminus is blue, and the C terminus is red. Note the predicted seven-transmembrane-spanning domains. (B) Genomic PCR of Neo3 and the flanking regions was used to show correct integration of the knockout cassette into the *G37* locus. (C) Gene expression was analyzed by RT-PCR. *G37* cDNA was used as a template with primers for G37, GPCR6, and RPL21 (an rRNA gene control). No *G37* expression was seen in the G37-KO mutant, but proper expression of the others verified the quality of the mRNA used. (D) Genomic PCR was used to identify ectopic G37 by using G37 and flanking pchxMTT primers. This showed that this could be used for both overexpression of wild-type *G37* (WT-OE) and rescue of the wild-type phenotype in the G37-KO mutant (G37-OE). (E) RT-PCR showed that WT *G37* is expressed in the rescued G37-KO mutant (G37-OE) following Cd^2+^ induction. The values beside the gels are molecular sizes in kilodaltons.

The protein structure predicted from the amino acid sequence of TTHERM_00633170 by GOModo is shown in Fig. 1A. While this does not prove that G37 is a GPCR, it does show that it can be fitted into a GPCR-like structure by a current program. Note the predicted seven-transmembrane-spanning regions. The *G37* gene (TTHERM_00633170) encodes a protein that shows some homologies with a well-described GPCR from *Dictyostelium* (the cyclic AMP receptor called CAR1) (26) and the previously described GPCR from *Tetrahymena* (GPCR6) (9). The percent identities to G37 are 26 and 28%, and the number of positives (sum of both identical matches and residues that have undergone conservative substitution) are 45 and 39 for CAR1 and GPCR6, respectively. This shows that there are some amino acid similarities between G37 and other protozoan GPCRs.

### Confirmation of the G37-KO mutant and overexpression constructs.

Biolistic transformation (12, 13) was used to deliver the *G37* knockout construct to the macronucleus of vegetative wild-type CU427 cells as previously described (9). The plasmid used to carry this construct was the same as that used for the GPCR6 mutant (9), except that the coding region of *G37* was substituted for the GPCR6-encoding gene. This *G37* knockout mutant (called G37-KO) was grown under increasing concentrations of paromomycin to allow phenotypic assortment and complete replacement of the endogenous *G37* gene with the Neo3 cassette. Genomic PCR confirmed the presence and successful recombination of Neo3 into the target locus by using primers specific to Neo3 and outer flanking sequences of the knockout construct (Fig. 1B). Loss of *G37* gene expression was verified by reverse transcription (RT)-PCR of *G37* cDNA (Fig. 1C). GPCR6 and RPL21 were used as RT-PCR controls to show the correct processing and stability of the isolated mRNA.

**FIG 2:**
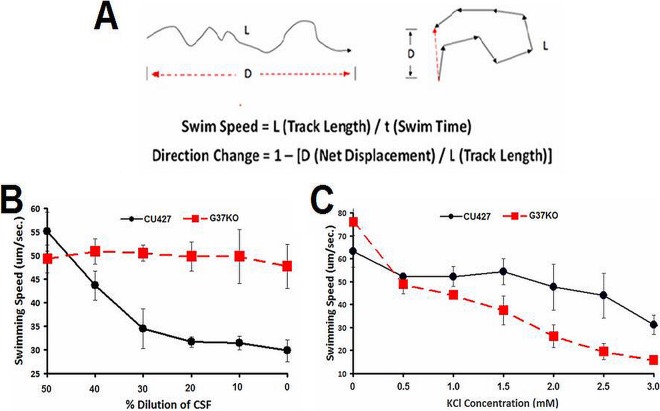
(A) Our Tetratracker program was used to measure the forward swimming speed of cells. Swimming speed was determined by measurement of the total length (L) of the path over time. (B) Wild-type CU427 cells decreased their forward swimming speed as the concentration of CSF increased, but the swimming speed of G37-KO mutant cells remained unchanged. (C) While both the wild type and the G37-KO mutant showed decreased swimming speed at high K^+^ concentrations, the swimming speed of the G37-KO mutant was lower than that of the wild type at the higher K^+^ concentrations. Each point is the mean ± the standard deviation of six different determinations of 50 to 100 cells by the Tetratracker program. *, *P* < 0.001 (Student *t* test).

To show that the wild-type phenotype could be rescued by the expression of wild-type *G37* in the G37-KO mutant, we created a *G37* overexpression (G37-OE) mutant (Fig. 1D). We also used this construct to analyze the effects of GPCR3 overexpression in wild-type (WT-OE) cells. In all cases, vegetative cells were biolistically transformed with a construct where the *G37* gene was placed in a Cd^2+^-inducible, ectopic expression plasmid (pchxMTTGFP, a gift from Doug Chalker). A map of this plasmid can be found at the Tetrahymena Stock Center webpage. This construct recombined into the RPL29 locus and conferred cycloheximide resistance on the transformants. RT-PCR confirmed the presence and expression of the ectopic *G37* gene in the G37-KO mutant (Fig. 1E). The various primers used for PCR are shown in Fig. S1 in the supplemental material[Supplementary-material figS2].

10.1128/mSphere.00182-17.1 FIG S1  Primers used for PCR analysis. Some of these were used to generate Fig. 1B to D. Download FIG S1, PDF file, 0.1 MB. Copyright © 2017 Zou and Hennessey. 2017 Zou and Hennessey This content is distributed under the terms of the Creative Commons Attribution 4.0 International license.

10.1128/mSphere.00182-17.2 FIG S2  Localization of the GFP-tagged G37 protein in the wild type. The pchxMTTGFP plasmid (a gift from Doug Chalker) was used for G37-GFP localization studies. Vegetative CU427 cells were biolistically transformed with a construct where the *G37* gene was placed in this Cd^2+^-inducible ectopic expression plasmid. This construct recombined into the RPL29 locus and conferred cycloheximide resistance on the transformants. RT-PCR confirmed the presence and expression of the ectopic GPCR3-GFP-encoding gene in the *G37* mutant. The *G37* gene was functionally expressed because it rescued the wild-type phenotype in the G37-KO mutant the same as the G37-OE. It is interesting that there may be localization to the base of the cilia because the orderly rows suggest alignment with the basal bodies. However, we need to do many more colocalization experiments to test this. (A) The black arrows show some of the cilia, but many of the others are outside this focal plane. (B) The white arrow shows the region of the oral groove (also out of the focal plane in panel A). Download FIG S2, PDF file, 0.1 MB. Copyright © 2017 Zou and Hennessey. 2017 Zou and Hennessey This content is distributed under the terms of the Creative Commons Attribution 4.0 International license.

### Swimming responses of wild-type and G37-KO cells to CSF and other chemorepellents.

We initially tested a number of known chemoattractants and chemorepellents (2) to see if the G37-KO mutant was defective in its response to any specific ligands. As a bioassay for chemorepellent behaviors, we used a computer-assisted program to analyze digital movies of the swimming paths of cells. This is a modification of the WRMtrck program used for analysis of the swimming tracks of *Caenorhabditis* cells with ImageJ (Jesper Pederson, personal communication) that we call Tetratracker (Fig. 2A). Since this assay reports on swimming path deviations from linearity, the DC is near zero when cells are swimming straight. Chemorepellents increase AR, CCR, and other DCs, causing an increase in the DC to up to 1.0.

We used this Tetratracker program to analyze the responses of G37-KO mutant cells to as many chemoeffectors as possible. The first thing that we found was that G37-KO mutant cells had altered responses to a CSF. Since this CSF was obtained by washing and incubating the cells in a dilute buffered solution, it represented a fairly simple fraction for our behavioral bioassays. Quantitation of these responses with the Tetratracker program showed that the swimming speeds of the wild type reliably decreased as the CSF concentration increased, while the swimming speeds of the G37-KO mutant cells were unaffected (Fig. 2B). The swimming speeds of both wild-type and G37-KO cells decreased as the K^+^ concentration increased, but this effect was more pronounced in G37-KO cells (Fig. 2C).

**FIG 3:**
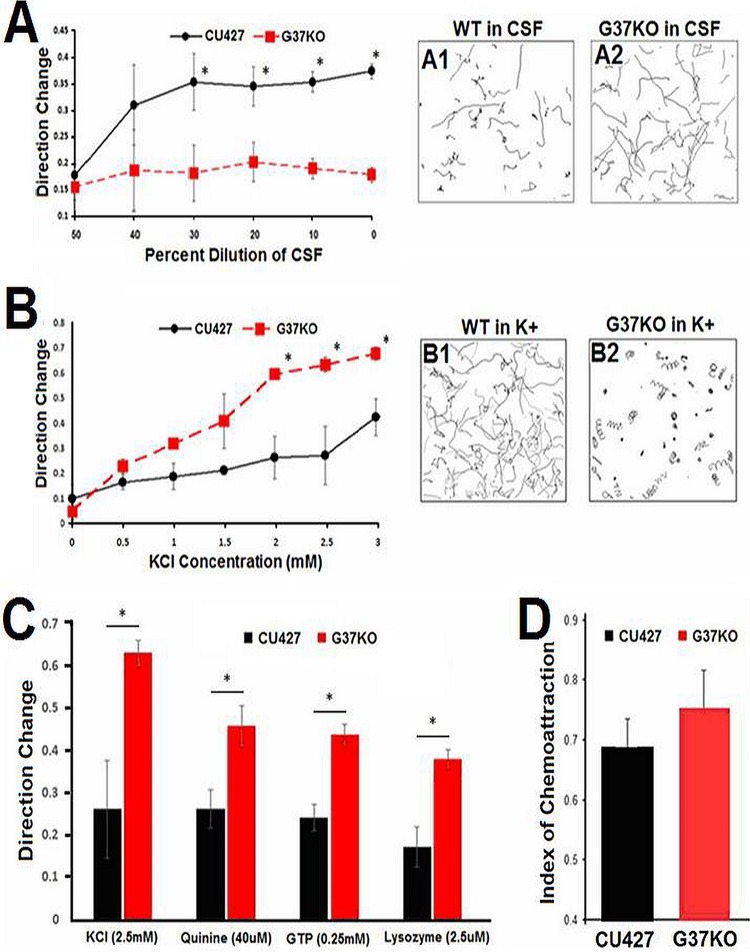
The Tetratracker program (see Fig. 2A) was used to quantitate chemorepellent behaviors. (A) The average DCs of wild-type (WT) CU427 (black line) and G37-KO (dotted red line) cells are shown. Wild-type cells responded to the CSF in a concentration-dependent manner, but G37-KO cells did not respond at any of the concentrations tested. One-second swimming paths of wild-type (A1) and G37-KO (A2) cells in 20% CSF are shown as representative images used for these analyses. (B) G37-KO mutant cells are overresponsive to high K^+^ concentrations. More DCs were seen in G37-KO (B2) than in wild-type (B1) cells at the higher K^+^ concentrations. Each point is the mean ± the standard deviation of six different determinations of 50 to 100 cells by the Tetratracker program. *, *P* < 0.001 (Student *t* test). (C) G37-KO mutant cells also overresponded to several other chemorepellents. More DCs were seen in G37-KO (red bars) than in wild-type (CU427, black bars) cells at representative concentrations of quinine, GTP, and lysozyme. Each data point is the mean ± the standard deviation of six different determinations of 50 to 100 cells by the Tetratracker program. (D) The wild type (CU427) (black bars) and the G37-KO mutant (red bars) show similar chemoattraction to 1.0 µM LPA in the classic three-way stopcock assay (42). An index of 0.5 represents no attraction, and 1.0 means 100% of the cells were in the test arm. Each point is the mean ± the standard deviation of three different determinations of cell counts in each arm of the three-way stopcock. *, *P* < 0.001 (Student *t* test).

Although the CSF is a powerful chemorepellent of wild-type (CU427) cells, G37-KO mutant cells are insensitive to it. While the CSF-containing fraction reliably changed the swimming behavior of wild-type cells in a concentration-dependent manner, similar to what would be expected from a chemorepellent, it had no effect on the swimming behavior of G37-KO test cells (Fig. 3A). At a 20% dilution of the stock CSF concentration, wild-type cells showed much more avoidance responses than G37-KO cells (in a Student *t* test, *P* < 0.001, *n* = 6 independent experiments).

We used the same behavioral assay to test the responses of G37-KO to a general depolarizing agent, a high K^+^ concentration. In stark contrast to its underresponsiveness to CSF, the G37-KO mutant overresponded to high K^+^ concentrations over the range tested (Fig. 3B). G37-KO mutant cells showed a significantly higher number of DCs than wild-type cells (in a Student *t* test, *P* < 0.0005, *n* = 3 independent experiments).

G37-KO mutant cells were not only overresponsive to high K^+^ concentrations, they were also overresponsive to all of the other chemorepellents tested (Fig. 3C). Comparisons of the wild-type and G37-KO responses in KCl, quinine, GTP, and lysozyme all showed significant differences between wild-type and G37-KO cells with all of these compounds. This suggests that G37-KO mutant cells have not only a specific defect in CSF sensing but also a general increased excitability in response to depolarizing stimuli.

There have been many reports of possible chemoattractants in *Tetrahymena*, but the only ones that we have been able to confirm as reliable chemoattractants are LPA (lysophosphatidic acid) (9) and proteose peptone (33). G37-KO mutant cells showed normal responses to LPA (Fig. 3D), further supporting the hypothesis that *G37* codes for a dedicated chemorepellent receptor.

### Expression of the *G37* gene in G37-KO rescues the wild-type phenotypes.

Since the absence of *G37* expression in G37-KO resulted in both underresponsiveness to CSF and overresponsiveness to other chemorepellents, we wanted to know if both of these phenotypes could be reversed (rescued) by ectopic expression of the wild-type *G37* gene in the G37-KO mutant. A Cd^2+^-inducible expression construct carrying the wild-type *G37* gene was used to transform CU427 and G37-KO to create WT-OE and G37-OE cell lines, respectively. Since cells were always washed before testing, this added Cd^2+^ (0.5 µg/ml) did not interfere with the behavioral assays. These cell lines were tested for their total avoidance response time (TRT). The TRT is the elapsed time from the start of avoidance swimming to the resumption of normal forward swimming to 20% CSF and 5.0 mM KCl. We used this manual TRT measurement because some of the conditions caused continuous backward swimming and this was difficult to quantitate by the Tetratracker program. This TRT measurement provided a more convenient way to make comparisons between groups and also provided a measurement of adaptation and deadaptation times.

**FIG 4:**
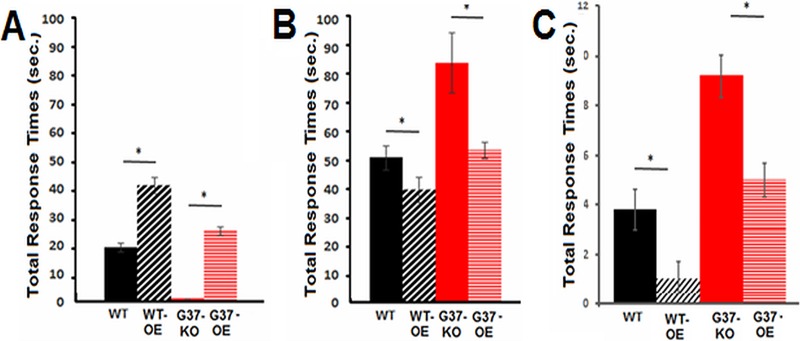
Effects of *G37* overexpression on responses to the CSF, high K^+^ concentrations, and a representative chemorepellent, lysozyme. The duration of backward swimming is expressed as the TRT. Data are shown for wild-type (WT) CU427 (black), *G37* overexpression in the wild type (WT-OE) (black slanted), G37-KO (red), and wild-type *G37* overexpression in the G37-KO mutant (G37-OE) (horizontal red). (A) Cells were tested in the undiluted CSF. Decreased response times indicate shorter overall durations of the response to the CSF. (B) Cells were also tested in 10 mM KCl. Longer response times indicate higher sensitivity to this depolarizing stimulus. (C) Cells were also tested for their responses to a representative chemorepellent, 20 µM lysozyme. Each data point represents the mean ± the standard deviation of 10 different experiments with 5 to 10 visual cell observations from Tetratracker images. *, *P* < 0.001 (Student *t* test).

While G37-KO showed no response to the CSF, expression of wild-type *G37* significantly increased the responsiveness of G37-OE (Fig. 4A). We also saw that WT-OE underwent even longer bouts of avoidance swimming in CSF than the wild type (Fig. 4A). Moreover, most of the CSF-induced avoidance swimming seen in WT-OE was the most extreme form, called CCR. Therefore, loss of expression of *G37* in G37-KO causes loss of responsiveness to the CSF while overexpression in WT-OE causes increased responsiveness to the CSF. Consistent with this, the loss of *G37* expression in G37-KO causes increased responsiveness to high K^+^ concentrations and overexpression in WT-OE causes decreased responsiveness to high K^+^ concentrations (Fig. 4B). As with the responses to CSF, the responses to high K^+^ concentrations were brought to wild-type levels by expressing wild-type *G37* in G37-KO mutant cells (G37-OE) (Fig. 4B). The response of G37-OE mutant cells to a representative chemorepellent, lysozyme, was also not different from that of wild-type cells (Fig. 4C). Taken together, these data strongly suggested that *G37* expression is directly linked to both CSF sensitivity and the general responsiveness to other chemorepellents.

### Adaptation to CSF caused decreases in responses to both CSF and K^+^ in the wild type.

**FIG 5:**
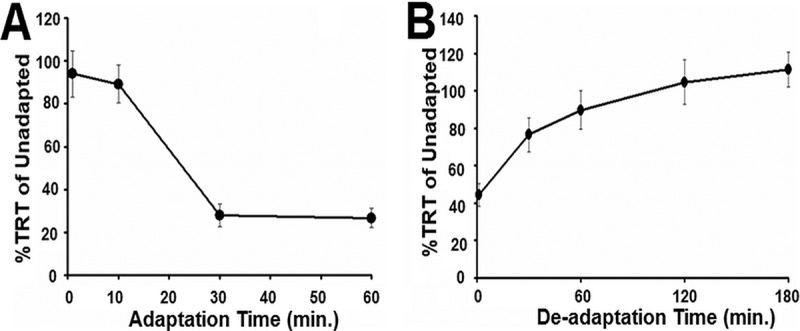
Behavioral adaptation and deadaptation to the CSF in the wild type. (A) TRTs of the wild type before and after exposure to the CSF were compared and are expressed as percent TRT relative to the unadapted (control) TRTs. After 30 min, the cells showed 20% of their original TRT. This decrease was due to adaptation to the CSF. (B) Cells were removed from the CSF after 1 h of adaptation and tested for the return of normal swimming. Each data point represents the mean ± the standard error 10 different experiments with 5 to 10 visual cell observations from Tetratracker images.

How could one gene defect (*G37* knockout) cause both a specific loss of response to the CSF and an overresponse to all of the other chemorepellents tested? This might happen if the CSF is always present in the growth medium, constitutively stimulating the wild type but not the G37-KO mutant. This could possibly lead to not only behavioral adaptation to CSF in the wild type but also cross adaptation to any other chemorepellents that use the same downstream response pathways. This general decrease in excitability would be seen in the wild type but not in the G37-KO mutant because the G37-KO mutant cannot sense the CSF. In comparison to the CSF-adapted wild type, the G37-KO mutant could show an overresponse to these compounds. We tested this hypothesis by adapting CU427 to the enriched CSF fraction (Fig. 5A). The cells were then washed and subsequently exposed to fresh CSF to measure their TRT. Incubation of the wild type in CSF for 1 h caused a significant decrease in the response to CSF upon retesting (Student *t* test, *P* < 0.001, *n* = 6), but since G37-KO cells do not respond to CSF, we could not use it to assay adaptation in G37-KO. Removal of the cells from the CSF resulted in deadaptation to the CSF (Fig. 5B).

**FIG 6:**
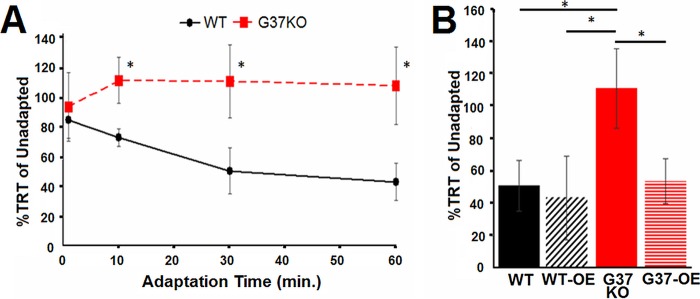
Cross adaptation to the CSF. (A) Behavioral adaptation of test cells to the CSF caused cross adaptation and a decrease in sensitivity to 10 mM K^+^ in wild-type (WT) CU427 (solid black line) but not G37-KO (red dotted line) cells. The percent TRT is a comparison of the TRTs of each cell line before and after 1 h of adaptation to the CSF. (B) Wild-type, WT-OE, and G37-OE cells were similarly affected by CSF adaptation, but G37-KO cells were not. Each data point represents the mean ± the standard deviation of 10 different experiments with 5 to 10 visual cell observations from Tetratracker images. *, *P* < 0.001 (Student *t* test).

The wild type also showed cross adaptation to high K^+^ concentrations after prolonged exposure to CSF, while the G37-KO did not (Fig. 6A). This cross adaptation to the CSF was also seen in the responses of the wild type to a representative chemorepellent, lysozyme. The response to 20 µM lysozyme was significantly decreased by a 60-min adaptation to 20% CSF (*t* = 34.1, df = 8, *P* < 0.0001). Expression of wild-type *G37* in G37-KO (G37-OE) caused a return to wild-type response levels, showing that cross adaptation between CSF and high K^+^ concentrations can occur in rescued G37-KO mutant (G37-OE) cells (Fig. 6B).

### Enrichment of the CSF-containing fraction.

Supernatants containing CSF could be isolated from 3- to 14-day-old *Tetrahymena* cultures grown at room temperature by the overnight incubation method. Both wild-type CU427 and G37-KO cultures yielded good CSF-containing supernatants, suggesting that G37-KO had no problem producing CSF.

**FIG 7:**
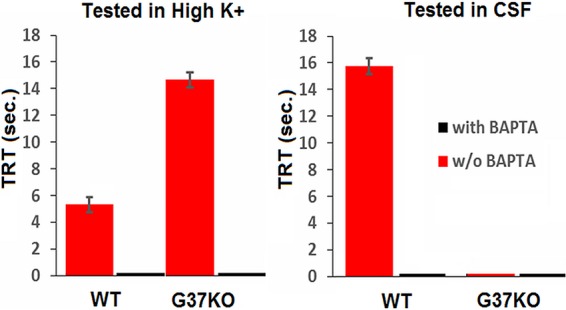
The responses of both the wild type (WT) and the G37-KO mutant were blocked by the Ca^2+^ chelator BAPTA. (A) The TRTs of the wild type and the G37-KO mutant in 5.0 mM K^+^ with and without BAPTA are shown. BAPTA blocked CSF-induced backward swimming in both. (B) The duration of the TRT in 20% CSF is decreased by BAPTA in both the wild type and the G37-KO mutant. Each data point represents the mean ± the standard error of three different experiments with 5 to 10 visual cell observations from Tetratracker images.

We have begun some preliminary analyses of the CSF to ascertain its identity. The CSF is unlikely to be a peptide because a wide variety of proteases failed to eliminate or reduce its chemorepellent effect and it is resistant to boiling. The spectroscopic properties of the CSF fraction indicated a broad local absorbance maximum at 255 to 260 nm (Fig. S3A). Although this spectroscopic pattern suggested the presence of nucleic acids, treatment with either RNase or DNase likewise had no effect on the CSF. This does not rule out the possibility of nucleic acid monomers, however. Since addition of 1.0 mM EGTA has been shown to eliminate both the behavioral and electrophysiological responses to a chemorepellent in *Tetrahymena* (34), we tested the effects of EGTA and 1,2-bis(*o*-aminophenoxy)ethane-*N*,*N*,*N*′,*N*′-tetraacetic acid) (BAPTA) on the responses to the CSF. External BAPTA (630 µM) eliminated the avoidance behavior of *Tetrahymena* in the CSF, suggesting that the activity of the CSF requires Ca^2+^ influx (Fig. 7).

10.1128/mSphere.00182-17.3 FIG S3  The absorbance spectrum of the CSF fraction and enrichment of the CSF. (A) The peak is around 255 nm, and there is no further absorbance in the visible range. (B) Separation of the enriched CSF. The crude CSF was separated on a Sephadex G25 column, and the enriched CSF eluted before the bulk of the material absorbing at 260 nm. The repellent activity was scored visually as 0 (no response), 1.0 (slow swimming), 2.0 (weak AR), 3.0 (repetitive AR), or 4.0 (CCR), and the average values of several observations were plotted. The repellent activity (orange line) was enriched in the earlier fractions containing OD_260_ absorbing material (blue line). Each image is a representative result of one experiment. Download FIG S3, PDF file, 0.1 MB. Copyright © 2017 Zou and Hennessey. 2017 Zou and Hennessey This content is distributed under the terms of the Creative Commons Attribution 4.0 International license.

We generated a further enriched CSF preparation by column chromatography. We found that this method reproducibly yielded an early peak in optical density at 260 nm (OD_260_) that contained almost all of the active CSF, according to behavioral testing (Fig. S3B). Since a *T. vorax* transformation factor called stomatin (31) was found by procedures rather similar to the ones we use to get the CSF, we also produced a crude stomatin fraction from our cells by using the previously published procedures. This stomatin fraction caused the same responses as our CSF and also caused transformation of *T. vorax* (not shown). This suggests two immediate possibilities. Either both the *T. vorax* transformation factor and our CSF are in this same fraction or they are the same compound. Further purification of the CSF should resolve this.

## DISCUSSION

The value of motile, eukaryotic, unicellular models like *Tetrahymena* and *Paramecium* for studies of intracellular sensory integration starts with the simplicity of the behavioral bioassays (2). Because it is well known that ciliary reversal and backward swimming are Ca^2+^-dependent events (15), AR and CCR can be used as indicators of high intraciliary Ca^2+^ concentrations. These effects can be verified by other traditional methods (1, 11), but the behavioral bioassays allow faster and easier first screenings (2) for biological compounds, drugs, and mutations that may affect receptor-mediated events. Ca^2+^ chelators such as BAPTA or EGTA can be used to verify the necessity for Ca^2+^ influx by lowering the external Ca^2+^ concentration (34).

Since we have identified a number of possible GPCR-encoding genes in the Tetrahymena Genome Database (9), we wanted to know if these predicted seven-transmembrane proteins are true GPCRs and what their functions are in *Tetrahymena*. Our approach has been to knock out the genes for these possible GPCRs and then look for the physiological effects of these knockouts. This is a reasonable approach because GPCRs and GPCR pathways involved in chemoattraction are well described in *Dictyostelium* (35) and knockouts of the genes for GPCRs that act as chemoattractant receptors have been previously described (36). Chemorepellents have also been described in *Dictyostelium* (27), but a chemorepellent receptor has not yet been identified. Our estimated structural and database analyses are all consistent with the idea that *G37* codes for a seven-transmembrane-spanning GPCR, but this does not prove that it is a GPCR. We have characterized the behavioral effects of the *G37* knockout, but identification of the ligand should be done before we can verify whether or not this is a GPCR, characterize the ligand binding, and study other possible downstream elements.

Verification of the *G37* knockout mutation required not only standard genomic and RT-PCR experiments but also rescue of the wild-type phenotype by wild-type *G37* overexpression in the G37-KO. This showed that the phenotypes were due to the intended gene knockout and not to any other alterations or mutations. To further confirm that the G37-KO phenotypes were due solely to the *G37* knockout, we also performed two more independent transformations of CU427 with the same *G37* knockout construct. These new G37-KO mutant cells showed no obvious differences from the original G37-KO transformants (data not shown). However, all of the experiments described were done with the original G37-KO line.

Because our original hypothesis was that GPCRs may serve as chemoreceptors in *Tetrahymena* (9), we screened as many possible chemoeffectors as we could to see if the G37-KO mutant had altered responses to any of them. The only thing that the G37-KO underresponded to was the CSF. Although we do not yet know what the active compound in this CSF fraction is, it is a considerably enriched and simple fraction. The responses of the wild type to this CSF are all consistent with its identification as a chemorepellent. The CSF caused decreased swimming speed and increased AR in the wild type but not in the G37-KO mutant. The G37-KO mutant is insensitive to the CSF.

To see if the underresponse of the G37-KO mutant to the CSF was due to a general loss of excitability, we used high K^+^ concentrations as a standard indicator of depolarization-induced ciliary reversal (2). This has also been commonly used as a bioassay for estimation of voltage-dependent ciliary Ca^2+^ channel activity in *Paramecium* because it provides the depolarization required to open the voltage-dependent ciliary Ca^2+^ channels (20). Contrary to what was seen in the CSF, G37-KO mutant cells showed DC overresponsiveness to high K^+^ concentrations. Consistent with this, G37-KO mutant cells showed lower swimming speeds than the wild type as the K^+^ concentration increased. This showed that the lack of responsiveness of G37-KO mutant cells to the CSF was not due to a general decreased excitability.

G37-KO mutant cells also showed overresponsiveness to all of the known chemorepellents we tested. This overresponsiveness was specific for chemorepellents because the responses of G37-KO mutant cells to the chemoattractant LPA were normal.

Since *G37* knockout caused a loss of responsiveness to the CSF but increased responses to the other chemorepellents, we wanted to see if overexpression of wild-type *G37* would normalize (rescue) these responses in G37-KO mutant cells. Overexpression of wild-type *G37* in G37-KO mutant (G37-OE) cells caused them to return to wild-type CSF response levels, and overexpression of *G37* in the wild type (WT-OE) caused an increase in the response of the wild type to the CSF. Similarly, the excessive high K^+^ concentration responses of G37-KO mutant cells were returned to nearly wild-type levels by overexpression of *G37* (G37-OE), while overexpression of *G37* in the wild type (WT-OE) caused a small decrease in the high K^+^ concentration responses. This showed not only the genetic linkage between underresponsiveness to the CSF and overresponsiveness to high K^+^ concentrations but also that the phenotypes caused by the *G37* knockout mutation were specific for the *G37* gene.

We found that chemosensory adaptation to the CSF not only caused a decrease in the response of the wild type to the CSF but also cross adaptation to high K^+^ concentrations. Cross adaptation to high K^+^ concentrations does not happen in the G37-KO mutant, consistent with its inability to detect the CSF. Furthermore, rescue of the wild-type phenotype in the G37-KO mutant (G37-OE) also enabled behavioral cross adaptation to the CSF because G37-OE cells could now sense the CSF. This supports not only the idea that *G37* is necessary for CSF adaptation but also the idea that this is not a specific CSF receptor event because cross adaptation to other chemorepellents is seen.

Chemosensory adaptation to the continued presence of the CSF may be the reason why G37-KO mutant cells are overresponsive to the other chemorepellents in comparison to the wild type. Since adaptation to the CSF appears to generally decrease the excitability of wild-type cells, their basal behavior in high-density cultures could be decreased because of the continued presence of the CSF. However, G37-KO mutant cells do not sense CSF and do not have this adaptation response, causing them to show greater basal excitability than the wild type. Since both the *G37* knockout mutation and chemosensory adaptation to the CSF cause changes in many different types of depolarizing responses, it is likely that all of these responses have something in common. All of these responses require Ca^2+^ entry through the voltage-dependent Ca^2+^ channel. Therefore, it is possible that one of the main actions of the CSF-activated G37 receptor is to modify the activity of this Ca^2+^ channel.

Changes in the properties of the voltage-dependent Ca^2+^ channels are one way to decrease the general excitability in these types of cells (20). The voltage-dependent Ca^2+^ currents of many neurons are inhibited by many different neurotransmitters and neuromodulators acting through their cognate GPCRs (37). Bean (38) suggested that inhibition of Ca^2+^ currents by GPCRs is due to a profound shift in the voltage dependence for channel activation with little or no effect on the number of functional channels. Since voltage clamp analysis has not been done with *T. thermophila*, we cannot address the possible effects of the CSF on Ca^2+^ currents at this time. However, the fact that BAPTA eliminates the response to CSF shows that Ca^2+^ entry is necessary, strongly suggesting a role for the voltage-dependent Ca^2+^ channels.

The only released factor that seems to be similar to our CSF is a compound called stomatin (31). Stomatin is found in the supernatant of *Tetrahymena* by using procedures that are very similar to our methods of obtaining the CSF (31). Stomatin causes transformation of the microstome form of *T. vorax* into the macrostomal form, which eats *T. thermophila* (31). One purpose of the CSF may be to act as a density-dependent chemorepellent to prevent *T. thermophila* from congregating at high enough densities to cause the transformation of carnivorous *T. vorax*. Avoiding high-density growth may also be advantageous to prevent overgrazing of bacterial food sources (39). When we prepared crude stomatin by the previously published procedure (31), we found that it was also active as a chemorepellent of the wild type, the wild type with *G37* overexpression (WT-OE), and the G37-KO mutant with *G37* overexpression (G37-OE) but not of the G37-KO mutant. Although this suggests that our CSF may be stomatin, it is also possible that the crude stomatin fraction contains both stomatin and our CSF. It is well known that *Tetrahymena* can release mucocysts and lysosomal enzymes into the growth medium and into a buffered solution (40, 41). However, the CSF chemorepellent is not coreleased with either of these because there is no difference between the CSF that is isolated from either mucocyst nondischarge mutant cells (41) or a mutant that does not secrete lysosomal enzymes (40).

An overall model that emerges from this work and the work with the G6 mutant (9) suggests that these two receptors (G37 and GPCR6) may represent opposing chemoattractant and chemorepellent systems. Together, these two receptors may help to set a baseline excitability level that can be either decreased or increased to cause either attraction or repulsion, respectively. GPCR6 may contribute hyperpolarizing influences from chemoattractants, and G37 can provide depolarizing inputs from chemorepellents. These might not be the only chemoreceptors in these cells, but they can be used to study the mechanisms involved in the integration of sensory information and consequent behavioral responses. Consideration of the changes caused by chemosensory adaptation may add plasticity to the process of deciding which way to swim in an open environment.

The major role that the G37 protein plays appears to be that of a chemorepellent receptor. These responses to the CSF may help cells to avoid certain areas in the environment such as overcrowding and overgrazing, as well as other, currently unknown, conditions. In this respect, *Tetrahymena* cells may use the CSF as an autocrine signal to communicate with each other. Since the CSF seems to be present in the medium at all times, chemosensory adaptation may also modulate the general basal level of excitability as the culture size and CSF concentrations change.

## MATERIALS AND METHODS

### Cell stocks, culture, and maintenance.

Wild-type *T. thermophila* (CU427.4) cells were obtained from Cornell University’s Tetrahymena Stock Center. All cells were cultured in 5 ml of SPP medium (1% proteose peptone, 0.2% glucose, 0.1% yeast extract) supplemented with 250 µg/ml penicillin G and streptomycin sulfate and 1.25 µg/ml amphotericin B. For overexpression mutant cells, the medium was additionally supplemented with 0.5 µg/ml CdCl_2_ for 24 h to induce gene expression and 50 µg/ml cycloheximide as a selective agent. Long-term stock cultures were kept in the above-described medium at 15°C, whereas working cultures were kept at room temperature (25°C). Cells were washed in wash buffer (5.0 mM morpholinepropanesulfonic acid [MOPS], 50.0 µM CaCl_2_ [pH 7.2] with Tris) prior to behavioral testing.

### Macronuclear knockout of *G37*.

Approximately 3.6 kb of DNA containing the *G37* (TTHERM_00633170) macronuclear sequence, as well as 1.2 kb of its upstream and downstream flanking sequences, was cloned into the pCR4-TOPO vector. The QuikChange site-directed mutagenesis kit (Stratagene) was used to introduce ClaI and BamHI sites near the 5′ and 3′ termini of the *G37* gene, respectively. The knockout construct was made by cutting the Neo3 antibiotic resistance cassette (gift from Martin Gorovsky, University of Rochester, Rochester, NY) from its vector with the introduced restriction sites and ligating it into the pCR4-TOPO vector to replace most of the *G37* gene. This knockout construct was then introduced into vegetative CU427 wild-type cells by biolistic transformation. Macronuclear transformants containing the knockout construct were cultured in increasing concentrations of paromomycin (up to 20 mg/ml) for about 2 months to completely replace the endogenous *G37* gene with the Neo3 cassette by phenotypic assortment. Complete gene replacement was confirmed by RT-PCR.

### Overexpression of wild-type *G37*.

The pChxMTTGFP plasmid (gift from Doug Chalker, Washington University, St. Louis, MO) was modified to remove the PmeI restriction site upstream of the MTT1 promoter, leaving only one PmeI site downstream of the MTT1 promoter. To do this, the original MTT1 promoter was cut from the plasmid with PmeI and replaced with a nearly identical MTT1 promoter insert that had an EcoRV site instead of a PmeI site upstream of the promoter. The *G37* overexpression construct was made by cutting out the green fluorescent protein (GFP) sequence with PmeI and XhoI and replacing it with the genomic GPCR3 sequence. All constructs were introduced into vegetative CU427 or G37-KO cells by biolistic transformation. Macronuclear transformants containing the construct were selected with cycloheximide (50 µg/ml).

### Preparation of the CSF-containing fraction.

About 0.5 ml of CU427 from a stationary culture was inoculated into 50 ml of SPP medium in a 500-ml flask. This was cultured at room temperature for 14 days. The 14-day-old cells were washed in wash buffer by centrifugation at 2,500 rpm, and then the pellet was resuspended in 50 ml of wash buffer three times. After washing, the cell pellet was resuspended in 5 ml of wash buffer and gently rocked overnight in a 15-ml conical tube. The overnight suspension was then centrifuged, and the supernatant was decanted into a fresh tube. The tube containing the supernatant was sealed and placed in a boiling water bath for 30 min to allow precipitation of unwanted compounds. After the heated supernatant was again centrifuged at 2,500 rpm to eliminate precipitants, it was loaded onto a 10-kDa concentrator (Pall Corporation, NY). This was centrifuged at 4,000 rpm for 1 h to completely pass the supernatant through the 10-kDa filter. The filtrate was the CSF-containing fraction. This is the fraction that was used in all of the experiments in this study.

### Behavioral testing.

Cells used for behavioral testing were grown in SPP medium for 2 days at room temperature. Prior to testing, cells were washed as follows. A total of 1,000 µl of cells was centrifuged in 1.5-ml microcentrifuge tubes at 7,000 rpm. A 20-µl volume of cell pellet was collected and suspended in 1,000 µl of wash buffer in 1.5-ml microcentrifuge tubes. The washed cells were kept at room temperature for 2 h prior to behavioral testing. Test solutions of KCl, quinine, guanosine 5'-*O*-[γ-thio]triphosphate (GTPγS), and lysozyme were prepared in wash buffer, whereas test solutions of the CSF were prepared in water. To begin behavioral testing, 5 µl of the test solution was pipetted into a single well of a 10-well polytetrafluoroethylene printed slide (Electron Microscopy Sciences, Hatfield, PA). A 5-µl volume of test cells was then quickly mixed with the test solution for approximately 4 s, and the resulting mixture was filmed with a Moticam 480 camera (MOTIC, Hong Kong) for 10 s at ×40 magnification. The behavior of test cells upon encountering the test solution was analyzed in the second, third, and fourth seconds of the 10-s video with a plugin for ImageJ that we call Tetratracker. Each cell track was analyzed for deviation from linear forward swimming paths, as well as path length. This information allowed calculation of the DCs and swimming speeds of multiple cells (usually about 60 at a time).

Cells for the chemoattraction assay were washed as described above and then tested in a three-way stopcock assay (9, 33, 42). One arm of the three-way stopcock contained the attractant (1.0 µM LPA), the opposite arm contained control solution (the wash buffer), and cells were added to the entry arm. The stopcock was opened for 30 min, and the index of chemoattraction was determined by counting the cells in each arm and calculating the percentage of the total number of cells in the test arm. An index of 0.5 represents no attraction, and 1.0 is the highest level of chemoattraction because 100% of the cells were in the test arm.

### Behavioral adaptation.

Test cells were grown in SPP medium for 2 days at room temperature and washed as described above. After 3 h of starvation at room temperature, 10 µl of the test cells was mixed with 10 µl of the CSF fraction or 10 µl of water for a specific period of adaptation (1 min to 1 h). A 980-µl volume of wash buffer was then added to the cells, and they were centrifuged at 7,000 rpm for 2 min. About 40 µl of the pellet was removed and placed into a fresh tube for testing. To test for cross adaptation to various repellents, 5 µl of 10 mM KCl, 80 µM quinine, 1 mM GTPγS, or 1 µM lysozyme was mixed with 5 µl of cells and the resulting mixture was filmed for 1 min with a Moticam 480 at ×40 magnification. The TRT of each cell was manually measured, and this was defined as the amount of time that elapsed until the cell resumed forward swimming after repellent addition. Deadaptation to CSF was tested by adapting test cells to the CSF for 1 h and then transferring them to wash buffer to be assayed over time in the wash buffer.

### Chemical treatments of the CSF-containing fraction.

The CSF-containing fraction was chemically treated by being mixed with various concentrations of pronase, proteinase K, RNase I, or DNase I for up to 8 h under manufacturer-recommended incubation conditions. Subsequent mixtures were directly tested on washed cells to determine if the avoidance effect of the CSF was abolished. The various enzymes were also tested on cells to determine whether they have any effect on the basal avoidance of *Tetrahymena*.

### Column fractionation and enrichment of the CSF.

About 0.5 ml of the crude CSF fraction was loaded onto a Sephadex G25 column (GE Healthcare, Chicago, IL) with deionized water as the liquid phase. Fractions of 0.75 ml were collected, and the OD_260_ was measured. Each fraction was tested for avoidance effects on washed cells. Fractions with noticeable avoidance effects were combined and concentrated to the original input volume (0.5 ml) by boiling.

### Size exclusion chromatography.

Molecular weight (MW) standards (blue dextran, myoglobin, and vitamin B_12_), uracil, and the active, concentrated CSF fractions from a Sephadex G25 column were sequentially loaded onto a fresh Sephadex G25 column with 0.1 M NaCl as the liquid phase. The elution profile of the MW standards was determined colorimetrically, whereas the elution profiles of uracil and CSF fractions were determined by measuring the OD_260_.
