# Supplementary material for: Behavioral Effects of a Chemorepellent Receptor Knockout Mutation in Tetrahymena thermophila
Source: mSphere. 2017 Jul 5;2(4):e00182-17. doi: 10.1128/mSphere.00182-17 (PMC5497023; doi:10.1128/mSphere.00182-17)
Supplement: FIG S1 [file sph004172316sf1.pdf]

G37 ORF + flanking region PCR Primers	
**Primer Name**	**Sequence (5'-3')**
G37ORF&FLK_5	TTAAATAGCTTTTGGAGAGATAGATAG
G37ORF&FLK_3	CTAGAAAAAGTTGTTTAGTATCTAATTTACATATTTA
Site-directed Mutagenesis Primers	
**Primer Name**	**Sequence (5'-3')**
G37_ClaI_SDM_5	GCCATTAGCATAATCGATGAGTTATGACGATTGTTTCTAACC
G37_ClaI_SDM_5	CAATCGTCATAAATCGATGCTAATGGCTTAATTCAAGCTT
G37_BamHI_SDM_5	CTCTGGCGAAGGCCAAGTTGAAATGGATCCACTTAATCAATGAACAA
G37_BamHI_SDM_3	GATTAAGTGGATCCATTTCAACTTGGCCTTCGCCAGAGGATAT
G37-KO Confirmation Primers	
**Primer Name**	**Sequence (5'-3')**
NEO3_5	AGAGGCTATTCGGCTATGAC
G37_OF3	GAAATCAAAGTGTTGGTCCATC
G37_cDNA_5	GCTCTTTTTCTCTTCTAGGAGCT
G37_cDNA_3	CCAGAGGATATGGATGCAGTT
GPCR6_5	GAGCTTTGATTACTTATGTCCCTT
GPCR6_3	GAGAGAAATCCGTAGACTATTGA
RPL21_5	GAGAGAAGTCAAGTCGCCAA
RPL21_3	GACGTTGCCTTCGAATTCGGT
G37 Overexpression Cloning + Confirmation Primers	
**Primer Name**	**Sequence (5'-3')**
G37MTT1_PmeI_5	CATGTTTAAACAAAATGACGATTGTTTCTAACCACA
G37MTT1_XhoI_3	CATCTCGAGTCATTGATTAAGTTGTTCCATTTCAACT
pMTTChx_5	CACGATTTATGCAATGATCCATATAAAAT
pMTTChx_3	GAATAGAAGTTTTGGTTGGTTGTTTGTT
