# Supplementary figures and images for: Behavioral Effects of a Chemorepellent Receptor Knockout Mutation in Tetrahymena thermophila
Source: mSphere. 2017 Jul 5;2(4):e00182-17. doi: 10.1128/mSphere.00182-17 (PMC5497023; doi:10.1128/mSphere.00182-17)

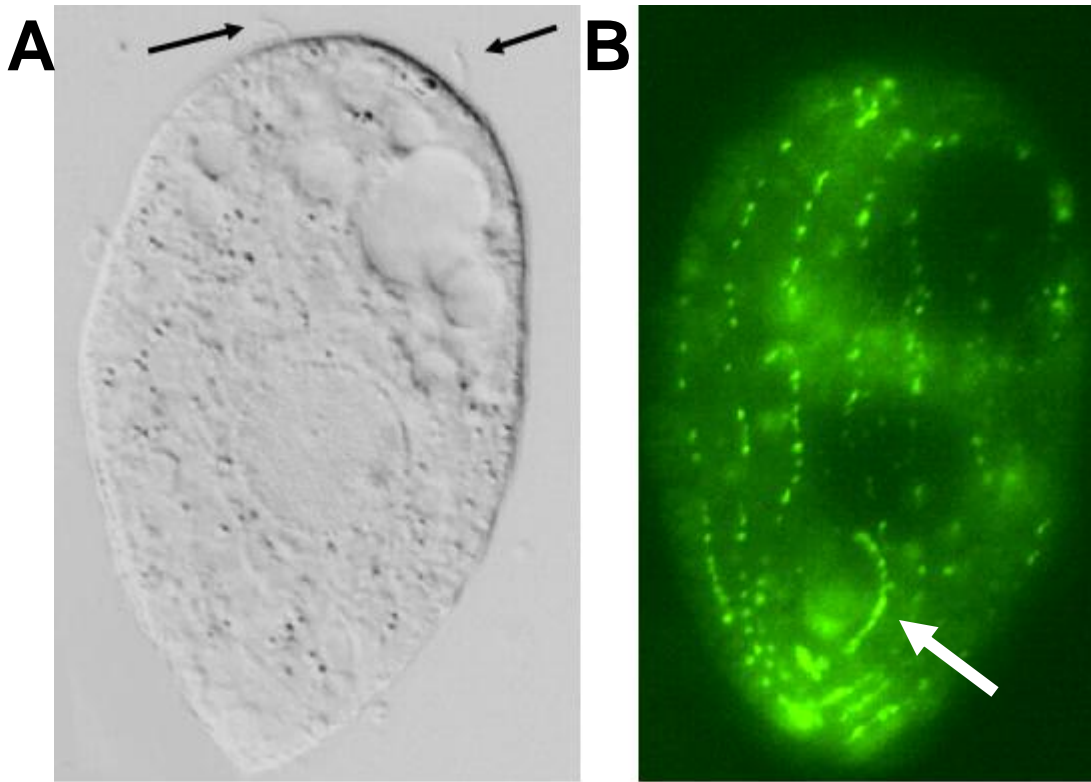

25um

Supplement: FIG S2 [file sph004172316sf2.pdf]

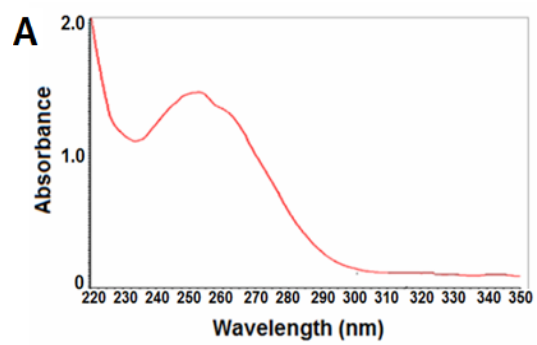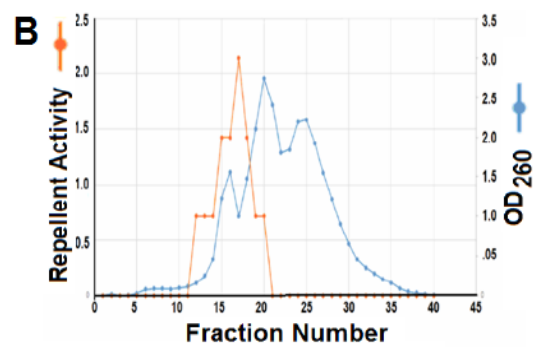

Supplement: FIG S3 [file sph004172316sf3.pdf]
